# Designing felt experiences with movement-based, wearable musical instruments: From inclusive practices toward participatory design

**DOI:** 10.1017/wtc.2022.15

**Published:** 2022-08-17

**Authors:** Doga Cavdir, Ge Wang

**Affiliations:** Center for Computer Research in Music and Acoustics, Music Department, Stanford University, Stanford, California, USA

**Keywords:** embodied music interaction, felt experiences, first-person experience, inclusive design, movement-based interaction, participatory design

## Abstract

Inclusive musical instruments benefit from incorporating wearable interfaces into digital musical instrument design, creating opportunities for bodily felt experiences and movement-based interactions. In this article, we discuss the evolution of our inclusive design approach behind the design and performance practices of three wearable musical instruments. By focusing on the embodied, somatic, and tacit dimensions of movement-based musical interaction, we evaluate these case studies, combining the third and first-person perspectives. The design and implementation of the wearable sensing, utilizing the additive manufacturing techniques, are discussed for each instrument and its performer in specific cases of musical expression. This article further discusses how our approach integrates music performance as a crucial step into design and evaluation, utilizing these performance practices and such collaborative settings for improved diversity and inclusion. Finally, we examine how our design approach evolves from user-centered design to more participatory practices, offering people with diverse abilities a shared music performance space.

## Introduction

Wearable technology (WT) has become significantly prevalent in today’s society. Designers integrate this technology into more common uses ranging from body-worn sensors such as assistive devices, smartwatches, or activity trackers (Zeagler et al., 2018; Tapu et al., 2020) to more artistic creations such as fashion design (Ryan, 2008; Hrga, 2019), dance performance (Katan, 2016; Kim and Landay, 2018; Nam and Kim, 2018; Giomi, 2020; Roth et al., 2021), or art for body awareness (Chappell et al., 2021). Similarly, musicians have been developing wearable electronics or adapting commercially available wearable devices for music composition and performance (Tanaka, 1993; Nymoen et al., 2015). Michel Waisfisz’s “The Hands” (Torre et al., 2016) and Laetitia Sonami’s “Lady’s Gloves” (Sonami, 2006; Reid et al., 2018) are early examples of custom-designed gestural controllers that are worn to capture hand and finger movements (Lai and Tahiroglu, 2012; Mitchell et al., 2012; Serafin et al., 2014). Due to their interaction methods, the performance space of these instruments is traditionally limited to small-scale gestures. Atau Tanaka’s WT detects bio-signals and involves on-body sensing in music (Tanaka, 2000). His Bio-Muse performance centers around the hand gestures that are less visible on stage due to EMG sensing. These wearable instruments engage in only a small gestural space, potentially losing visual aspects (feedback and communication) of an embodied performance. Body movements convey important visual and kinesthetic information, reflected on the musical expressivity (Bahn et al., 2001; Dahl and Friberg, 2007). As Dahl and Friberg explain, visual perception of music-related body movements significantly contributes to the connection between the performer and the audience (Bahn et al., 2001; Dahl and Friberg, 2007). Similarly, in this research, we focus on the connection between performers and audience members with three wearable musical interfaces engaged with dual gestural vocabulary. To visually and kinesthetically amplify the performance and listening experiences, these wearables merge the gestural interaction with expressive body movements. Their gestural vocabulary combines nuanced musical gestures with dance-like larger body movements. During a performance, these instruments deliver an embodied listening experience to the audience while simultaneously emphasizing the felt, bodily experience of music-making for the performer (Engelsrud, 2005; Höök et al., 2018; Mah et al., 2021).

Contrary to glove-like musical controllers, Tomie Hahn and Curtis Bahn’s collaborative performance piece, Pikapika, extends the gestural space by coupling the musician’s body with wearable devices beyond on-body sensing, such as wearable speakers and amplifiers (Hahn and Bahn, 2002). Hahn’s performance visualizes a full-body interaction captured by wearable electronics and embodies the sonic interaction by amplifying sound directly with the “body-mounted speakers” on each arm. Similar to Pikapika, more recent dance–music interfaces explore dance interaction using full-body movement (Camurri, 1995; Camurri et al., 1999; Mason, 2012). Although this approach delivers the full-body movement and visual aspects of music performance, it requires a collaboration with an external musician or composer. This performance practice divides the roles of dancer/choreographer and musician/composer. To provide more musical autonomy to the dancer and to offer more embodied performance opportunities to the musician, we suggest holistically bringing two perspectives closer with movement-based musical instruments. This approach prioritizes the first-person experience of the performer by extending the instrument with the performer’s body (Höök et al., 2018).

Ceraso (2018) studies how embodied listening contributes to more inclusive music-making. Among inclusive design studies, wearable musical interfaces are often researched to support the experiences of disabled musicians or listeners (Frid, 2019). Integration of body movements and felt experiences is often little studied in the inclusive design of wearable, movement-based musical interfaces, specifically research addressing diverse hearing abilities. Soma-based design uses the designer’s lived body as a resource in the design process to highlight the first-person approach (Höök, 2018). Some research on somaesthetics discusses how to effectively communicate physical discomfort and limitations, including sharing the experiences of disabled users that are “highly personal and difficult to discern from outside” (Beuthel and Wilde, 2017). In addition to incorporating embodied design practices, users’ participation contributes to improved accessibility and inclusion in musical interface design (Muller, 2003; Caramiaux et al., 2015). Oswal (2014) emphasizes why participatory design approaches are central to designing systems built on accessibility and how accessible and usable designs are possible through direct involvement of participants. Quintero (2020) studies how participatory design of experiences through collaboration and codesign not only “establishes communication alternatives for people with disabilities,” but also develops a holistic understanding of participants’ motivation and rehabilitation needs in real-life situations. In our research, participatory design practices provide us with the opportunity to include the performers as the designers through their lived, bodily experiences. Additionally, because music is an embodied language and body movements support disambiguate musical features (Dell’Anna et al., 2021), centering this instrument design practice around movement-based interaction helps improve the hearing impaired users’ perception and experience of music, providing collaboration among people with diverse abilities. We draw from these embodied, soma-based, and participatory approaches to design movement-based, wearable musical instruments for more inclusive listening and music-making, utilizing WT to incorporate body movement on a larger scale while maintaining the tangible interaction. In addition to offering more embodied listening practices, these wearable instruments offer felt experiences of music performance (Cavdir and Wang, 2020). Our approach to delivering embodied musical experience contributes to the design of three inclusive instruments for diverse bodies, presenting a shared space between music and dance.

Through three case studies, this study analyzes the embodied, felt experience of the performer and the audience members. First, we develop wearable musical instruments that not only capture nuanced musical gestures but incorporate expressive body movements. These instruments are extended by the performer’s body; in other words, they are considered incomplete without their performers. Combining different gestural levels from nonmusical practices supports developing bodily awareness for musicians and allows the dancers to leverage their existing background and experience in movement-based practices. This gestural vocabulary also communicates visual and kinesthetic information to the audience while delivering felt listening experiences. Second, using these wearable instruments, we bring the roles of musicians and dancers closer in the same performance space. Finally, we apply WT to more embodied performance and listening for diverse bodies. Our evaluation focuses on studying users’ creative outcomes and performance practices by developing a shared performance space. We utilize this shared space across either different artistic backgrounds or diverse abilities to create more inclusive music performances.

A roadmap to the rest of the paper: section “Background” presents a brief overview of WT used in music performance and inclusive music-making. In section “Design Approach and Methodology,” we present our design approach and discuss how this approach incorporates implementation and performance considerations of wearable electronics. Section “Case Studies” individually details three case studies including the wearable interface designs, user studies, and results from the qualitative analyses. In section “Emerging Themes and Practices,” we synthesize emerging themes across three case studies and their creative artifacts. In section “Discussion,” we discuss how to design felt experiences using wearable musical instruments, how to develop better inclusion in music performance by creating shared performance spaces, and how our design approach is evolving from user-centered design to more participatory practices. This section also examines how qualitative studies, emphasizing the first-person approach, are fundamental and evaluate participants’ experience with movement-based wearable instruments.

## Background

### Critical Lens on Wearable Musical Instruments

Wearable musical instruments have been researched throughout the development of digital musical instruments (DMIs). These technologies were initially developed as hand or glove controllers by musicians, specifically to create customized interfaces and compositions such as Waisfisz’s “The Hands” (Torre et al., 2016), Sonami’s “Lady’s Gloves” (Sonami, 2006), and Tanaka’s Biomuse (Tanaka, 1993). The wearable interfaces were also employed in interdisciplinary music performances, consisting of sensor-integrated clothing (Ryan and Salter, 2003). This research discussed the tangible relationship between the participant’s improvised movement and musical response. Similarly, Pikapika explored full-body interaction in musical interface design and performance (Hahn and Bahn, 2002). By incorporating full-body wearables, these research projects extended the performance space of the musical interaction. Although they focused on full-body movements of the performers, the design process distinctly divided the designer and participant roles. For example, Pikapika involves an interactive collaboration between a dancer and a musician for the composition where musical features are divided between two agencies without providing the dancer with full control of music-making.

Although recent research studies new interfaces for inclusive music-making and developing assistive devices for people with disabilities, the study of wearable musical instruments for inclusive design remains limited (Frid, 2019). While some studied the vibrotactile stimuli through different modalities with wearables (e.g., bone conductance) (Trivedi et al., 2019), some researchers focused on assisting hearing-impaired users with embedding haptics on clothing such as vests (Novich and Eagleman, 2014) or delivering music information using on-body sensing with a haptic chair (Jack et al., 2015). These designs offered hearing impaired other modalities to receive music information. However, only a few of these designs offered tools for creating music, primarily because wearable musical interfaces design research provided users with little or no opportunities for participation.

### Embodied Musical Interaction

Drawing from Merleau-Ponty’s (1962) theory of *phenomenology of perception* that opposes the separation of body and mind, the embodied interaction explores how bodies and technologies might interact with each other. Dourish (2004) discusses this notion in the human–computer interaction (HCI) context to reintroduce the social, emotional, and physical aspects of interaction and draw designers’ attention from sole cognitive approaches. Similarly, Klemmer et al. (2006) develops five interaction design themes around the body as the center of experiences, understanding, and interactions in the world. These design approaches have been reevaluated in forms of movement-based interaction design or designing for kinesthetic experience (Moen, 2006; Loke and Robertson, 2013) to highlight the body’s central role in experience and engagement with the world. Additionally, Spiel (2021) highlights the connection between embodied interaction and increased diversity in design considerations.

Music literature includes numerous approaches that emphasize embodied design. Earlier work addresses disembodiment issues that computer–music interaction introduces, such as “the loss of intimacy between human player and instrument” or “instrument form that is decoupled from function” (Cook, 2004; Wang, 2018. Through embodied cognition and interaction design specifically in music performance and expression, Leman (2008) emphasizes that the body is central to our experience of music. Although he highlights such bodily experience (an experience that is directly felt and understood through the body and body movement) is an integral part of music, his studies on musical gestures emphasize the interaction with the instrument and the performance environment and exclude body movement or movement sensation from music interaction.

A relevant body of research that focuses on embodiment in interaction design and highlights *the body as the musical instrument* extends from the biosignal-driven interaction to nontactile (air-gestures) musical performances. Tanaka and Donnarumma (2019) explore the idea of the body as a musical instrument through electrical and mechanical muscle activity detection. Another perspective on the *body as instrument* derives from the theory that designs interactive systems for musicians’ kinesthetic awareness (Mainsbridge, 2016). Similar to Mainsbridge’s nontactile interaction design, yet more focused on traditional musical interactions, Godøy et al. (2005) study air-instruments–nontactile performance of traditional musical instruments. Similarly, Nijs and Leman focus on the interaction between musicians and traditional musical instruments and adopt the opposite approach to Tanaka and Mainsbridge’s work. They discuss how instrument becomes *a natural extension of the body* (Nijs et al., 2009).

### Inclusion, Diverse Bodies, and Participation

As Beuthel (2020) emphasizes, “the human body is a manifestation of diversity.” When working with wearable musical interfaces, the design process requires awareness and understanding of others’ abilities and skillsets. Inclusion in design ranges from accessible design to designing for all (Keates et al., 2000). While some of these design approaches focus on addressing specific groups or disabilities, others create shared and accessible environments for all, considering the broadest spectrum of user abilities (Samuels, 2015). Samuels and Schroeder (2019) study how technologies serve for more inclusive applications if the designers adopt a bespoke design, driven by “the unique strengths and abilities of an individual user.” This bespoke design approach not only offers more inclusive musical instruments but also enables designers to develop more accessible interfaces as well as performance spaces for all. Considering the unique abilities of an individual contributes to the inclusion of a wider community of disabled users and musicians. Similarly, Spiel (2021) echoes the importance of centralizing diverse and marginalized participant voices in design and in relation to technology, aiming to *understand individuals’ unique viewpoints.* Addressing this challenge of inclusion in design necessitates active participation of users beyond serving as subjects of normative research (Bannon et al., 2018; Spiel, 2021).

Frid et al. (2019) contribute to inclusive sonic interaction design by providing multiple modes of interaction in an interactive sound installation. Although the researchers focus on a general user group instead of addressing needs of specific disabilities, they provide more accessible interaction for diverse bodies. Lucas et al. (2019) discuss the use of diverse personas in inclusive design practices and the challenge of addressing the needs of “atypical” abilities of a user group. Some researchers explore addressing individual needs through increasing users’ active participation in the design process. For example, Dickens et al. (2018) examine the methods to participate the users into the DMI design process in real-life interactions, grouping the instruments into four categories of tangible, gesture, virtual, and wearable. Similarly, Lucas et al. (2020) conducted a participatory design study for the design of accessible musical interfaces, by collaborating with a disabled artist. Although few studies emphasized the inclusive and participatory design practices, a small number of design research investigated these practices in wearable musical instrument design.

Inclusive practices are crucial for participatory design research and in return, participatory design or codesign practices support increased inclusion. Wright et al.’s (2010) stage model of participation presents that inclusion prepares the design practice for increased participation. Similarly, Duarte et al. (2018) synthesizes the participatory design and research approaches and report important principles that are also parallel to inclusive and accessible design practices. They discuss the importance of *defining community and its empowerment* in participatory design. Unger (2013) highlights this practice of augmenting participants’ knowledge beyond sole involvement by offering training and workshops and by providing participants with ways to reflect on their practice.

## Design Approach and Methodology

In our design approach, we investigate three musical interfaces and performance practices in three case studies. Each of the three studies involves one interface design, evaluation of the interaction and the wearable system, and cocreating creative artifacts engaging with participants of diverse hearing abilities and/or artistic backgrounds over two years.

The first case study investigates how movement-based interaction influences music-making through transferring choreography practice into composition practice. The embodied, body- and movement-based music practice creates new applications in inclusive music-making. One such application offers more bodily listening experiences for both hearing-impaired and hearing audiences. This application is evident in our second case study, which focuses on incorporating nonmusical communicative gestures into wearable instrument design and creating shared listening experiences. Using the instrument from the second case study, we more actively collaborate with hearing-impaired individuals in the third case study. This study emphasizes the participation of hearing-impaired movement artists in the design of wearable haptics for music. Our codesign practice is informed by some of the key elements of participatory design outlined by Halskov and Hansen (2015): diverse participation, mutual learning and generation of key concepts, and iterative actions. The development of a movement-based music making practice in the first two case studies support a shared, inclusive, and safe design and performance space in the third case study to include hearing impaired artists in music and cultivate mutual learning through codesign.

The three case studies strongly highlight the first-person perspective of the performer (a combined role of the musician and the mover) and emphasize incorporating the performer into the design process as a cocreator. Drawing from soma design and embodied music interaction, we employ three tactics: (a) defamiliarization, (b) extending musical movement vocabulary, and (c) creative artifacts (Van Der Schyff et al., 2018; Höök et al., 2021).

We apply the defamiliarization tactic in both the instruments’ wearable design and gestural interaction and movement vocabulary. Because DMIs introduce new musical interactions, they often provide unfamiliar interfaces and gestural vocabularies in music-making. Throughout the three case studies, this tactic is strongly present when incorporating an unfamiliar, nonmusical gestural vocabulary such as dance movement or sign language gestures. When working with wearable electronics, we frequently utilize defamiliarization with technology. The relationship between familiar and unfamiliar is balanced by using sonic interaction from traditional musical practices (e.g., string sound) or familiar felt sensations of sound vibrations (e.g., feeling bass frequencies from subwoofers).

Secondly, through introducing nonmusical gestures and body movements into music-making, we employ a soma tactic into musical interaction, which is extending one’s movement vocabulary. Contrary to performing with traditional musical gestures, we encourage participants to explore new sound-producing and sound-facilitating gestures (Godøy and Leman, 2010) by focusing on the movement composition. This interaction can take the form of a single new gesture or combined into gestural motifs or gestural sequences. It can also extend to using different body poses or body’s spatiality Larssen et al. (2006), which are less frequently explored in traditional instrument practice. The third tactic focuses on creating music and movement artifacts as ways of (a) learning the instrument, (b) evaluating the interaction, and (c) producing research outcomes. Creative artifacts are produced in different modalities, such as music, movement, or both, combining the two artistic practices. This tactic is employed in different ways but always combines music and movement, such as creating a musical statement using a movement-based musical instrument (section “Bodyharp”), performing an inclusive and embodied listening space (section “Felt Sound”), and cocreating music-movement vocabularies that are communicated through haptic interaction between performers (section “Touch, Listen, and (Re)Act”).

These three tactics are used in each case study throughout the research, leading users to participate in new ways of music-making and listening using body movement. With explorations of sound through interactions of the moving body, we can share underlying embodied listening practices and reveal how codesign might facilitate a shared creative space.

### Process

All three case studies followed four stages to study and evaluate the interaction: (a) designing musical interfaces through movement-based interaction, (b) engaging participants with the musical interfaces or compositions, (c) cocreating artistic artifacts, and (d) collecting participants’ self-reflections. This practice-based approach encouraged participants to more intentionally approach music-making through body movement, build movement awareness of their movement interaction in order to replicate the sonic and physical gestures, and utilize improvisation in music composition and movement choreography beyond exploratory practices.

The interview and questionnaire data were transcribed after each study and thematic analysis was independently conducted for each case study. Each study and their data analysis followed the following procedure:

- Collect data: The user demographics and responses to the experiences were collected in a combination of entry and exit questionnaires and interviews. In addition to collecting qualitative data, the compositions were internally recorded using the Chuck programming language^1^ and externally recorded using a Zoom audio recorder.
- Create artistic artifacts: The studies investigated the process of either learning or creating a movement-based instrument to compose musical and movement statements as an artistic outcome. These creative artifacts were sometimes composed by participants (the first case study), performers (the second case study), or codesigners (the third case study).
- Make a content log: We reviewed the demographic information of participants and summarized their backgrounds and expertise.
- Transcribe: We selected data to transcribe and annotate.
- Analyze Themes: We identified common themes that emerged from both the questionnaire and interviews. We also included some of our third-person observations from participants’ movement interaction. This third-person analysis was important specifically when working with participants with little prior music knowledge or perception (i.e., the first and third case studies) or with participants who lack the vocabulary to articulate movement qualities (i.e., the second and third case study).
- Reflect and discuss design implications: We iterated the designs for the next case studies, reflecting on participants’ interaction and creative expression toward their more active participation.

Some case studies included additional steps in the procedure (such as follow up performances in the first case study or public demonstration in the third case study). The individual study themes were narrowed down to common, emerging themes across three studies. These emerging themes are reported in detail in section “Discussion.”

#### Approaches

##### First case study

The first case was examined through a user study, firstly leading the participants to learn, explore, and create with the instrument and its affordances. The participants individually developed new music and movement compositions with the interface after practicing with it. The study is followed by collecting participant feedback and self-reflection through questionnaires and semi-structured interviews. Oral consent was collected before the user study and each participant was compensated for the 2-hr session. The study and the interviews were audio–video recorded. The interview recordings were deleted after completing the transcription and the video recordings were deleted after analyzing movement data.

##### Second case study

The second case study utilized the same approach to music-making through nonmusical gestures as the first study but did so in an immersive sound environment where the participants were encouraged to interact with the sound sources to amplify the bodily sensations of listening. This study progressed through three short performance sessions involving a group of participants as the audience. The participants voluntarily joined the performance. At the beginning of each session, participants were provided with the context of the performance, which involved a low-frequency, high amplitude composition and emphasized a shared listening experience for diverse hearing abilities. The performance was only recorded for documentation purposes and did not capture the audience. Participant feedback was anonymously collected in writing.

##### Third case study

Movement-based music-making and embodied listening practices were combined in the last case study to include the participants as codesigners throughout the process. This study included three workshops and one public performance and demonstration session. Three workshops were organized to iteratively codesign wearable haptic interfaces and collaborative music-movement performances. Each workshop concluded with a discussion on the haptic, music, and movement interaction and by collecting designers’ reflections that were only audio recorded for transcription. The resulting composition was presented in a public performance and the haptic interfaces were demonstrated to the participants of the public session. Their reflections were collected through an exit questionnaire and a discussion. Due to pandemic-related restrictions, the collaborative performance was documented virtually with the consent of the performers.

#### Participants

##### First case study

Following the IRB approval for nonmedical human subject studies, the first case study recruited 20 participants from Bay Area local artists and authorized in-person researchers from Stanford University’s CCRMA. The participants were invited via email and were provided with the information sheet and oral consent form when they arrived. Participants were selected primarily from artistic practices of music, movement, or both. Although no music or movement background was required, all participants reported that they had multidisciplinary artistic backgrounds or practices.

Table 1 presents the demographic information (age group [Age], music [Music] and movement [Movement] experiences in years and their dominant practices [Dominant]) of the participants in the first case study. These experiences are based on their reported experiences and range from amateur self-practice to professional levels. The study asked participants to perform the same tasks regardless of their prior experience.

**Table 1.** Participant demographics of the first case study

P	Age	Music	Movement	Dominant	P	Age	Music	Movement	Dominant
1	35–40	1	35	Movement	11	45–50	30	30	Movement
2	50–55	2	15	Movement	12	30–35	10	11	Music-Movement
3	25–30	10	5	Music	13	20–25	14	0	Music
4	25–30	5	16	Movement	14	20–25	22	12	Music-Movement
5	45–50	41	6	Music	15	25–30	25	1	Music
6	30–35	27	0	Movement	16	20–25	15	3	Music-Movement
7	70–75	—	—	—	17	35–40	30	0	Music
8	65–70	30	0	Music	18	20–25	20	3	Music
9	20–25	20	22	Music-Movement	19	20–25	18	6	Music-Movement
10	—	—	—	Music	20	35–40	26	0	Music

##### Second case study

The second study was conducted with eight participants as audience members over three performance sessions in a special immersive performance space, Listening Room, at Stanford University’s CCRMA. Participants were invited via email and they provided their oral consent before proceeding. Participants had considerable music training with an average of 18+ years and familiarity with new musical interface performance. They had no reported hearing impairments. Seven participants communicated with spoken English and had no background in signing English and only one communicated primarily with both speaking and signing.

Table 2 presents participants’ age group (Age), music experience (Music), how often they move to music (Movement), hearing impairments (Hearing), and experience with performance using American Sign Language (ASL). None of the participants experienced hearing impairments and some had experience with performing arts that uses ASL either as artistic material or for increased inclusion of presenting the performance to the deaf communities. All participants had prior experience in music. Their music experience in years is indicated in parenthesis.

**Table 2.** Participant demographics for the second case study

P	Age	Music	Movement	Hearing Impairments	ASL
1	30–35	Piano and voice (25)	Always	None	None
2	30–35	Piano (27)	Often	None	ASL performance
3	30–35	Piano and flute (20)	Not often	None	None
4	20–25	Drums and trumpet (10+)	Always	None	None
5	20–25	Voice (10+)	Always	None	None
6	25–30	Multiinstrumentalist (20)	Always	None	Song interpretations
7	25–30	Piano and percussion (20+)	Sometimes	None	ASL theater
8	25–30	Jazz guitar (7)	Often	None	None

##### Third case study

In the third case study, the first workshop started with three participants, two of whom joined in person (P1 and P3) and one virtually (P2). The workshop series was conducted with the support of ShareMusic & Performing Arts Center^2^ in Malmö, Sweden. This study focused on codesigning haptic interfaces and music-movement performance with one participant (P3) who is profoundly deaf and has a dance background. The music and movement composition was supported by another participant (P2) who has a background in physical theater and no reported hearing disabilities. P1 decided against continuing the workshop series after the first session. The rest of the workshop and performance series as well as the evaluation of the study was continued by actively involving P3 in the design process. The Deaf participant (P3) communicated with Swedish Sign Language and equally associated with both hearing and Deaf/Hard of Hearing communities. The sessions always included two Swedish Sign Language interpreters and P3’s assistant whenever P3 was present.

Table 3 presents the participants’ age group (Age), hearing abilities or impairments (Hearing), cultural associations with hearing and/or Deaf/Hard of Hearing (D/HoH) communities (Cultural Association), and communication languages (Language). One hearing participant (P1) communicated with both signed and spoken languages, one hearing participant (P2) communicated with only spoken languages, and one deaf participant (P3) only communicated with signed language. Both P1 and P2 reported their interest in the project because they had relatives with hearing impairments. Majority of participants equally associated with both hearing and D/HoH communities.

**Table 3.** Participant demographics for the third case study

P	Age	Hearing	Cultural association	Language
1	40–45	Hearing	Mostly with hearing	Signed, Spoken Swedish, Spoken English
2	45–50	Hearing	Equally with hearing and D/HoH	Spoken Swedish, English
3	40–45	Profoundly deaf	Equally with hearing and D/HoH	Signed Swedish

#### Experiment designs and setup

##### First case study

In the first case study, a six-step experiment asked participants to learn the instrument through guided exploration and create artistic outcomes. The first four steps encouraged participants to learn the gesture-to-sound mapping and develop a movement vocabulary by exploring: (a) the instrument with no sound feedback, (b) the string interface and its corresponding sounds using larger body gestures, (c) the hand controller, plucking strings, and the sonic response using nuanced gestures, and (d) a combination of both gestural domains. The final two steps asked participants to create an artistic performance with and without the instrument by: (e) composing a short musical statement and (f) performing a free movement improvisation in response to their composition. They reflected on their first-person experience with these creative processes (composition and choreography) on the questionnaire and in the interview. The preconceived themes were developed based on core research considerations, experiences gathered during the project, Bodyharp’s earlier prototype, and study design. The procedure was led following the individual elements from data collections to creative outcomes and performance.

This experiment’s last two steps focused on learning a new interaction pattern through creating music and movement compositions, revealing the underlying correspondences of these two interaction modalities. These steps highlighted the performer’s first-person experience with the moving body while focusing on music-making. Drawing from this bodily experience of the performer in the presence of sound, we applied a similar approach to bodily listening in the second case study.

The participants were provided the information sheet, study description, and oral consent form at the beginning of the experiment. All participants gave their oral consent to audio–video record them for transcription, data analysis, and academic presentation purposes.

##### Second case study

The second study investigated how visual and kinesthetic elements of music performance were received by the audience and how such performance offers an increased inclusion to music. Because the physicality of the listening experience was at the core of this study, the performance included a gesture-based music composition performed live and the audience was encouraged to interact with the subwoofers. Each session briefly introduced the concept of sign-language-based, low-frequency, and high amplitude composition to the audience. Later, the composition was performed through an 8-channel subwoofer speaker array surrounding the audience. The audience was encouraged to sit close to and touch the speakers.

The performance sessions were held with small groups of audience members (3–4 participants each) for them to interact with the sound sources. Before each performance, a short introduction to the performance context was provided and participants’ oral consent was collected. All participants volunteered their consent us to audio–video record them for research and presentation purposes and use their anonymous data for data analysis. After each performance, participants’ demographic information and responses to the experience in an exit questionnaire were collected in writing. Data collection was followed by an open discussion with participants of each session which was audio recorded and included in the thematic analysis of their experiences.

##### Third case study

By combining movement- and body-based interaction methods and embodied listening practices, the third case focused on a more participatory approach for codesign with Deaf participants. Before starting each workshops, we collected participants’ oral consent to audio–video record them for research and presentation purposes and use their anonymous data for data analysis. Building on the second case study, the first workshop in this third case introduced the previous project and the objectives and directions. This introductory meeting served as a survey to collaborate with Deaf individuals or their relatives and to recruit codesigners. At the end of the first workshop, we collected the participants’ demographic information such as their hearing, cultural associations, and sign and spoken languages as well as their music listening, performing, and movement or dance practices, both orally and in writing. After this workshop, one Deaf dancer was recruited for the codesign and coperformance research.

The second workshop focused on cocreating haptic interfaces with the Deaf dancer to explore on-skin haptics for different musical pieces and sound effects. The workshop tested two modalities of haptic feedback (in-air and on-skin), positioning the on-skin actuator prototypes, and P3’s experience with different musical compositions. The study participants first listened to four different sound files with a two-subwoofer array and later listened to the same sound files with a prototype of a wearable haptic module. The first part of the experiment tested how perceptive the participant was to different musical instruments and qualities when listening to the piano, singing, African drumming, and compositions from the second case study with two subwoofer speakers where the speakers were facing the participants on the left and right sides. In the second part of the experiment, the same test was repeated with the haptic module. In addition to exploring how these music pieces were perceived on-skin, we also tested the wearability of the modules. In this workshop, the Deaf dancer participated in the codesign of the wearable module to explore its design, material, and locations on the body.

In the last workshop, the designers cocreated a music-movement mapping and performance with the Deaf dancer. A listening practice used the second prototype of the haptic modules. This workshop focused on creating a collaborative performance between the two codesigners—the Deaf dancer and the hearing musician. The mapping between music and dance gestures was created based on the gestural vocabulary developed in the second case study and the dancer’s movement repertoire. In a remote session, codesigners’ reflections were collected and the documentation of the dance movement vocabulary was recorded.

The workshop series concluded with a public performance and demonstration session. This session started by presenting the development of the project and the performance created by the codesigners. The second part of the session offered a demonstration so the participants could experience the listening with the in-air and on-skin haptics. The participants reported their listening experience and reflections on the performance in an exit questionnaire.

### Evolution of Design Approach

Throughout the three case studies, our design and study approach evolved from user-centered approaches to participatory design. This change developed significantly as we studied the applications of movement-based interaction to inclusive design in music. The accessibility and inclusion aspects of the third study necessitated blurring the boundaries between the researcher, designer, and user. As Sander states, how users “want to express themselves and to participate directly and proactively in the design development process” became central in order to address specific needs of and collaboration possibilities with Deaf participants (Sanders and Stappers, 2008).

The first two case studies prepared the design and performance work for such participation due to their strong emphasis on *design for experiencing.* Both studies combined methods to obtain *explicit knowledge*, *observable information*, and, with a stronger emphasis, *tacit knowledge* by reversing the schema Sanders (2002) describes. To access the tacit knowledge that cannot “readily be expressed in words” (Polanyi, 1983), we initially asked participants to express their musical ideas through designed experiences and by constructing creative artifacts (such as listening experiences, musical statements, or movement compositions). By observing their interaction and creative outcomes, we identified the emerging themes and explored these themes through qualitative methods. In order to access explicit knowledge, we encouraged participants to verbalize their experiences and reflect on both their interactions and creative processes. Although our design methods shifted to more participatory methods, constructing creative artifacts as research outcomes remained integral to all three studies, emphasizing performers’ first-person experience during movement-based music-making.

Building on these three access points, participatory practices in the third case study also allowed us to extend this framework to “reveal *latent needs*, that is, needs not recognizable until the future” (Sanders and Stappers, 2008), which became especially effective in codesigning with participants with specific hearing needs and assistance requirements. Making listening to music accessible through body-based technology revealed the Deaf participants’ expectations from assistive music technology in directions that offer direct access to performance and composition. The coperformance stage in our design with the Deaf dancer derived from a similar motivation to more actively involving her in the participatory process. As an artifact, collaboratively creating a mapping between dance and music gestures offered an understanding of intuitive relationships between the two, supported listening beyond solely auditory practices, and created new potential for the hearing impaired to create with music.

Additionally, all studies provided a new experience that infers what the participants think. In other words, the experience breaks the expectations from music and movement practices through an unfamiliar interaction. This exchange between familiar elements of music or movement performance and unfamiliar interactions or unconventional listening practices was achieved with the exploration of body- and movement-based creativity while utilizing technology. This creative practice reveals underlying associations and relationships between two domains and simultaneously avoid using the technology as a specific tool; instead integrating it in the process of expression. Addressing these associations and relationships become more crucial when designing with participants who have different levels of music experience, understanding, and perception. For example, the third case study focuses on a codesign for music performance with a participant with little music experience and perception due to hearing disabilities or similarly, the first case study investigates musical interaction of participants with two different artistic background and naturally different levels of music knowledge. The balance between the familiar and unfamiliar can offer more inclusive entry points to movement-based music-making.

Moving forward, in this research, we are aiming to evaluate the potential of movement-based musical interaction for more inclusive performance practices through codesign. Additionally, the case studies are structured toward increasing awareness on body movement, embodied interaction, and listening, emphasizing performers’ felt experiences.

## Case Studies

### Bodyharp

#### Interface design

**Figure 1.**
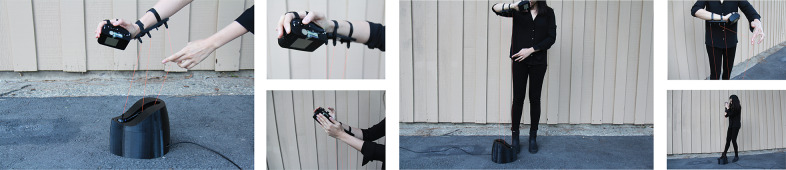
Bodyharp consists of two wearable interfaces (attachment to the performer’s arm and a hand controller) and a main instrument body.

The Bodyharp consists of an instrument body and wearable parts, including an attachment to the performer’s arm and a hand controller. Figure 1 presents the Bodyharp’s most recent 3D printed interface components, showing the wearable hand controller enclosure with tactile sensors, wearable arm-string attachment piece, and the instrument body enclosing the string-pulley system. The connection between the instrument and the wearable parts completes the interface by integrating the performer’s body, thus the instrument cannot be considered without its performer (Cavdir, 2021). This hybrid system, combining WT with the human body, offers new embodied ways of designing musical instruments and considers the instrument and the body as extensions of each other. (Nijs et al., 2009; Mainsbridge, 2016; Cavdir, 2021).

Our approach to Bodyharp’s design incorporates embodied musical expression (Leman, 2008) and movement-based interaction (Loke and Robertson, 2013). It employs the design consideration that drives from simultaneously capturing nuanced musical gestures and large-scale body movements. We approach this concept by coupling the performer’s gestures with a wearable interface at two levels of body movements. Larger scale movements contribute to kinesthetic and visual aspects of the performance. They exude a dance-like quality that invites embodied skills and somatic expressions to be transferred into music performance. Smaller-scale gestures offer a nuanced control over musical events that are captured by more tactile sensors. Their interaction focuses on finger or hand gestures in a smaller periphery.

Same interaction principles are followed in two iterations of the Bodyharp’s interface. The performer starts interacting with the instrument by plucking or stretching the strings and continues by controlling the parameters with finger gestures. The sound excitation starts with playing individual strings and is followed by adding sound effects by larger arm movements. In the first iteration, the exoskeleton detects smaller-scale gestures and in the second iteration, the hand controller allows the performer to control the parameters of these sound effects. Similarly, larger body movements, either captured by the strings or by the accelerometer, change these parameters while simultaneously extending the musician’s performance space. These movements provide more freedom in space and expression, indirectly controlling but influencing the music.

#### Design approach: Body as an extension of the instrument

**Figure 2.**
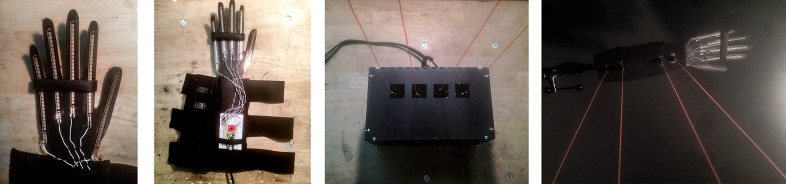
Bodyharp’s first prototype included an exoskeleton and an instrument body. The exoskeleton in the second iteration is replaced by the arm-string attachment and hand controller.

Bodyharp’s both prototypes focus on extending the musical interface with the performer’s body, directly incorporating their arms, hands, and torso as integral parts of the interface. In this design, the performer’s body acts as an extension of the musical interface and correspondingly the musical interface extends the musician’s body (Cavdir et al., 2018; Cavdir and Wang, 2020). The first design of the instrument includes a wearable arm piece and an exoskeleton (see Figure 2). The arm piece encapsulates the controller system and the accelerometer to detect the performer’s arm movements and to map them to sound parameters. The exoskeleton worn inside of the hand extends from the arm and detects finger movement with a series of flex sensors. The data from the flex sensors are similarly mapped to more nuanced controlled sonic events. This initial interface also includes the main instrument body that holds the string-pulley system, allowing the strings to extend from the instrument body and connect to the performer’s body.

The second design iteration addresses the design considerations of Bodyharp’s control mechanism and the technical challenges of the first prototype. In the first prototype, five flex sensors failed to provide a wide range of control for nuanced gestural performance and sound control. They also lacked passive force-haptic feedback since they only rely on the bend of fingers in a limited range. These sensors additionally offered less reliable data and more variance between mappings in each performance as their deflection profile and consistency change over their life cycle.

To address these challenges, we replaced the exoskeleton with a hand controller. The finger-bend interaction is replaced with buttons on top of the controller. Two force sensors are added: the first is accessible with the thumb on the right hand wearing the instrument, and the second is accessible with either the freehand, other body parts (chest, legs, arms, etc.), or the environment. We observed performers engaging with this second sensor using their chest, legs, arms, or heads. This additional sensor, facing outwards from the performer’s body, improved the performer’s interaction with their bodies and their environments. Similarly, the accelerometer, placed inside the hand controller, is relocated from the forearm to the palm. Changing the accelerometer’s location created new possibilities for hand gestures. In its earlier position (on the forearm), the sensor was able to capture the orientation and movements of the arm in a limited range. In its final position (on the palm), the controller can still detect the arm orientation and movements, but it can also extend these affordances with shaking and waving hand gestures. By capturing the performer’s hand gestures, our final design extended Bodyharp’s movement vocabulary.

#### Results: Movement supports sonic interaction

In these two studies with Bodyharp (Cavdir et al., 2018; Cavdir, 2021), we explored how body movement contributes to the sonic interaction when it is used as a sound-facilitating and sound-modifying gestures (Godøy and Leman, 2010). Similar to the first prototype, the second iteration maintains the interaction mechanism of dividing sound excitation and nuanced control of sound effects and parameters. In this second prototype, the sound is created by the performer’s interactions with the strings (plucking, stretching, or moving the attached arm). The sound effects are solely controlled with hand or finger gestures on the hand controller.

In a user study with the second iteration (Cavdir, 2021), participants commented on these nuanced, small-scale gestural interactions to control the sound effects and parameters. Most participants stated that the interaction with the strings and the touch sensors was the most effective part of the gestural interaction. They reported that the touch (FSR) sensors provided a “nuanced dynamic control.” Their responses showed that force-sensitive control created a tactile interaction during which most participants engaged with both gestural interaction and sound creation.

In addition to the hand controller, interaction with the strings was reported to be intuitive, engaging, and effective. Table 4 presents their self-reported experiences as participants responded to touch sensing and string interaction. Strings enabled participants to interact with both small-scaled gestures, such as plucking the strings, and with body movements, such as dynamically controlling their kinesphere and instrument-body shape. One participant emphasized the effectiveness of the dual gestural interaction by highlighting this combination of interaction with (a) musical gestures that directly influenced the sounds and (b) body movements that allowed investigation by being more elusive. Similarly, another participant commented on two levels of interaction: “I could alternate between using the whole-body, big, and emotionally charged movements and smaller, more delicate gestures.”

**Table 4.** Participants’ self-reported experiences with the tactile interaction of the wearable interface are presented

Themes	Quotations
Question 1: *What was the most effective part of the interaction?*	
Touch sensing	“Having nuanced dynamic control (FSR sensors) with more fuzzy control of pitch/drone allows me to get into the sound/feel part of the system”
Tactile sensing	“I liked the buttons especially, the pulsating effect”
String interaction	“I loved the plucked part! I could feel the instrument reacting to me and I reacted to it”
String and touch sensing	“I really liked the intuitive nature of the controls, particularly with the strings and the palm and the pressure things”
Strings and touch sensing	“I enjoyed the range across gain, FSR vibrato sensitivity [… and] the quieter nuances […] Also, the gracefulness of the strings”
Question 2: *What gestural/movement vocabulary did you engage in?*	
Touch sensing	“Having nuanced dynamic control (FSR sensors) with more fuzzy control of pitch/drone allows me to get into the sound/feel part of the system”
String interface	“Shaking the strings!”
String interface	“The physical string pulling, and plucking was very engaging. Great interaction”
Question 3: *Describe your experience creating/controlling sound through body movements*	
String interface	“Bodyharp […] has a strong physical component to it due to the space its strings transverse. Quite graceful and also powerful”
String interface	“I liked the kind of restrictions the Bodyharp had. The long arm sweeps for long and plucking for short notes”

*Note.* Participants referred to the *touch sensing* as FSR, pressure, and touch sensors, they commented on the buttons and sliders, grouped under *tactile sensing*, and they described their experiences with the *string interaction* using plucking, stretching, string keywords.

All participants explored their space use and range of movement. Regardless of participants’ backgrounds, they all reported their relationship with Bodyharp’s space and shape affordances:

- “Not every instrument allows one to stretch the limbs. This one creates an ambiance that allows the body to be playful.” (space and shape)
- “…I can frame my body around it.” (shape)
- “It was a joy to explore the sonic space while moving my body in the physical space.” (space)
- “…Bodyharp similarly has a strong physical component to it due to the space its strings transverse. Quite graceful and also powerful.” (space)

Extending the performer’s use of space and movement vocabulary is crucial to offer an embodied music interaction in the Bodyharp’s design which necessitates a combination of wearable and free interaction with body movement. The instrument is not limited to a wearable interface that the performer acts on but merges both the interface and the body, both of which simultaneously react to each other. The Bodyharp’s wearable interface bridges the tactility of instrumental gestures and embodiment of the full-body movement interaction. This combination provides tactile interaction through touch on strings and sensors, creating opportunities for nuanced control over the sound that resembles musical gestural interaction. The bodily lived experience is built by physically incorporating the body (the performer’s arm) into the musical interface to form a single instrument as a whole through the performer’s movement, force interaction, and resistance from strings and touch.

#### Synthesis: Shared perspectives

Bodyharp encourages performers to leverage their artistic practice while developing awareness in either music or movement. In our user study, we worked with participants with various artistic practices, ranging from music performance, composition, and design to dance, choreography, performance arts, and poetry. Our goal was to investigate these artists’ varying perspectives and analyze differences and commonalities in how they interact with movement-based musical instruments. As a research outcome, the study led users to construct creative artifacts. As we explored both movement and music interaction, participants were asked to create a music composition and a choreography based on their own musical statements.

The results of our user study revealed that Bodyharp’s movement-based interaction provides a shared perspective to participants with musical and movement backgrounds. Based on the participants’ demographics and their common interaction patterns, we analyzed their response to Bodyharp from two perspectives: musicians and movers. Because musicians are traditionally trained to focus on the dexterity of instrumental gestures and nuanced control over the sound, when playing Bodyharp, they focused more on expressing articulate musical ideas and performing with musical gestures than they were with nonmusical body movements. However, many participants with music backgrounds reported that “the Bodyharp helped them to feel less self-conscious about their expressive body movements when they performed with the instrument compared to the free movement practice.” Similarly, participants with movement backgrounds translated their experience in dance and choreography into music-making despite little or no experience in music performance or composition. They reported that “they utilized their knowledge of choreography techniques to create a sound composition that is choreographed through movement interaction.”

Despite different backgrounds and expectations, musicians and movers reported shared experiences after performing with the Bodyharp. The instrument provided an introduction to each other’s practices. While Bodyharp offered a more embodied practice for the musician, it provided a more accessible musical interface for the mover, who already practiced a prior movement expression. Through qualitative questionnaires and interviews, we collected some of the shared experiences. Both musicians and movers shared that (a) Bodyharp was intuitive and encouraged them to explore new movement possibilities, (b) learning Bodyharp’s musical features based on its gestural affordance was effective, (c) Bodyharp enabled creating music through nuanced gestural interaction and body movements, and (d) physical constraints created new artistic possibilities.

**Figure 3.**
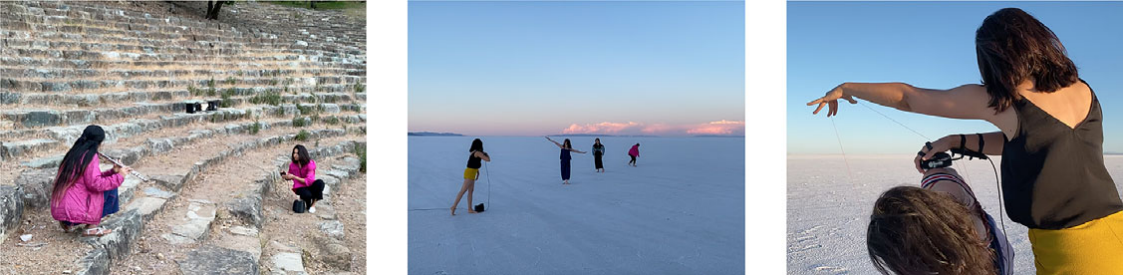
Bodyharp’s performance practices were developed based on the participant’s feedback and reflections, showing (a) a duo performance with a flutist, (b) a quartet with three dancers, and (c) an interactive duo performance with a dancer.

Lastly, we asked participants how they imagined Bodyharp to be used in performances. Many of them reported that it can be played as a collaborative or duo performance. The performance settings included collaborative dance and theater pieces, duo performances with dancers, musicians mimicking dancers in interdisciplinary settings, and accompanying other musicians in ensembles. One participant with a dance and education background suggested using Bodyharp as a movement-based art piece supports “the creative process in returning to a place of not knowing” in dance teaching. Some of the feedback from participants were employed in real-life performance situations. Figure 3 shows three case studies of performance with Bodyharp, following the results of the user study: Bodyharp was played in (a) a duo performance with a flutist, (b) a quartet with three dancers where dancers used Bodyharp’s movement patterns as cues for choreographic events, and (c) a duo performance with a dancer, interacting together with the string and touch sensors.

### Felt Sound

#### Interface design

Felt Sound is a musical interface that incorporates ASL gestures into music performance. The Felt Sound’s performance is designed to offer an embodied, felt listening experience for Deaf and Hard of Hearing (D/HoH) audiences (Cavdir and Wang, 2020) by combining low frequency and high amplitude in-air vibrations with physical gestures inspired by ASL. This instrument incorporates nonmusical communicative gestures (Godøy and Leman, 2010) to musical features that are composed to create physical sensations. The physicality from both the vibrotactile sensations as well as the gestural performance offers a shared, bodily, and felt listening experience for both D/HoH and hearing listeners.

In designing this accessible digital musical instrument (ADMI), we addressed two design considerations: (a) employing sound waves played through a subwoofer speaker array as a tactile feedback modality and (b) embodying ASL-inspired gestures for wearable interface design and musical expression. The overarching design objective draws from creating an inclusive music performance that offers an embodied listening modality for D/HoH and hearing listeners and provides a music composition that brings both audience groups’ experiences closer. This shared listening experience affords interaction with the subwoofer speakers, allowing the listeners to sit closer to the speakers. The audience members are encouraged to touch the speakers and sense the musical changes through their bodies when they simultaneously receive visual feedback of the gestural composition.

The musical interface maps the musical events to the gestural vocabulary of ASL-inspired finger, hand, and arm movements and nuanced gestural control. The gestural vocabulary consists of five ASL signs, performed in semi-structured improvisations (Table 5). The gestural compositions leverage ASL gestures’ communicative and expressive functions to provide a musical context to the D/HoH audience. Our motivation behind these compositions incorporates an unconventional gestural vocabulary outside music into the DMI design. Such design necessitates a wearable interface centered around the specific gestural vocabulary.

**Table 5.** Felt sound’s American sign language-inspired gesture-to-sound mapping

ASL gesture	Meaning	Sound engine	Detection
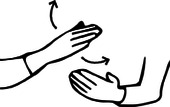	Music	Low-frequency beating	Acceleration
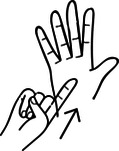	Show	Trigger drones	Magnetic sensing
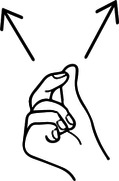	Poetry	Frequency change	Pressure sensing and acceleration
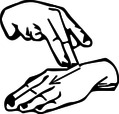	Empty	Clear all sound engines	Magnetic and pressure sensing
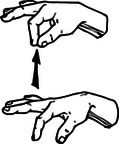	Discover	Add a new sound engine	Magnetic and pressure sensing

#### Design approach: Modular design

The wearable interface is modularly designed to allow designers and performers to customize the gestural interaction. The separate modules capture varying levels of gestures: nuanced finger gestures, single-hand gestures, and small arm gestures from both hands interacting with each other. The interface includes fingertip modules, passive elements like magnets for magnetic sensing, an accelerometer, and a controller module. These modules are prototyped using additive manufacturing where some parts such as magnets or accelerometers are embedded into the modules during the 3D printing and can be combined as desired on the left and right hands, wrists, and fingers (Cavdir, 2020). The fingertip modules detect finger interaction with a hall effect sensor triggered by a wearable magnet and force-sensitive resistors (FSR) for more continuous control. While the FSR and hall effect sensors are fixed on the 3D printed fingertip structure, the wearable magnet can be placed in multiple places, such as on the palm, on the back of the palm, or on the wrist, depending on the desired gestures.

**Figure 4.**
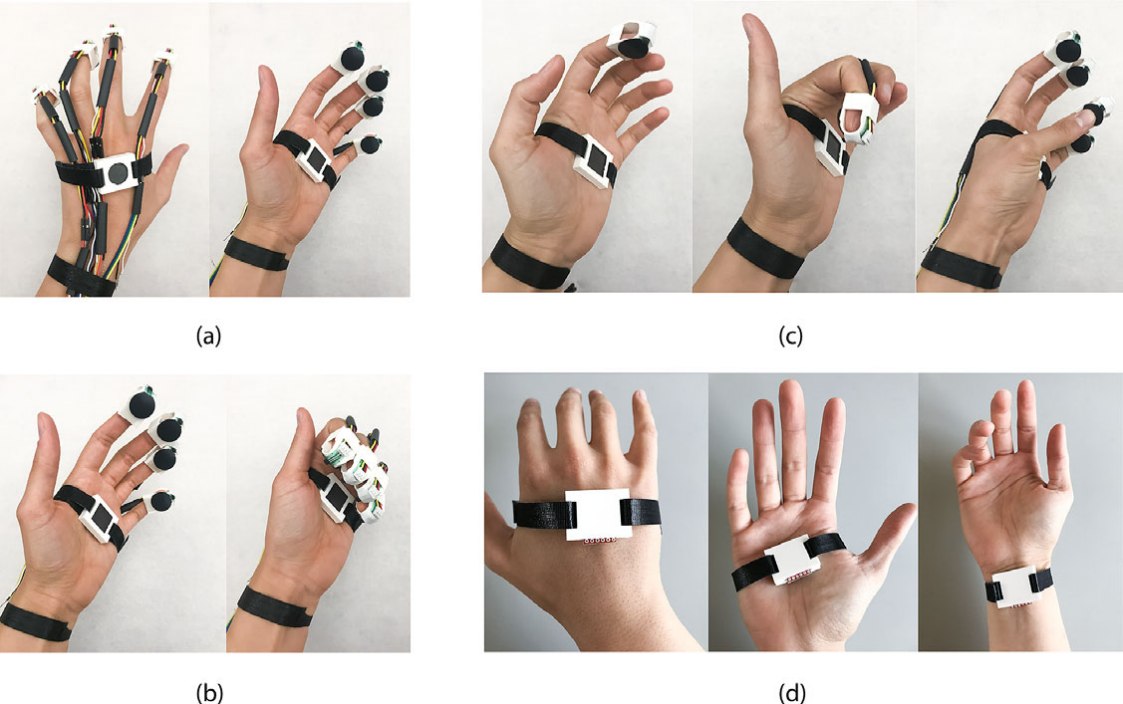
Felt Sound’s first prototype: (a) All modules are worn on one hand, (b) Fingertip sensor and a magnet module interaction change the state of the hall effect sensor while the FSR sensors output continuous data to control frequency, gain, and filter and effect parameters, (c) The hall effect sensors and the magnet placed on the palm allow detecting first closing and opening gestures, and (d) Accelerometer module embedded into the 3D printed closure can be placed in multiple locations on the hand or wrist and coupled with other modules.

Since the detection mechanism is limited to available sensors, the modular design creates flexibility to customize gesture-to-sound mapping. For example, single finger interaction can be extended to multiple fingers to create fist opening and closing gestures (see Figure 4b,c). Similarly, more dynamic movements, captured by the accelerometer module, can be worn in multiple locations to detect different ASL signs (shown in Figure 4d). The modular design captures the “show” and “empty” signs with the same group of sensors at different locations in the hands.

**Figure 5.**
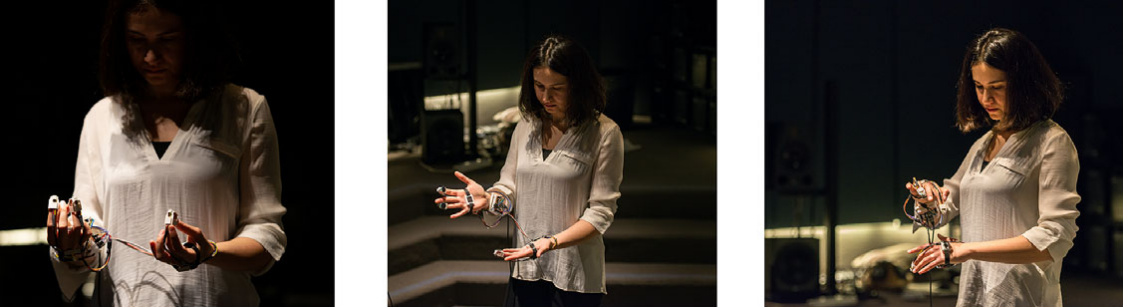
Felt Sound’s performance with sign language-inspired gestures, showing “music,” “poetry,” and “empty” gestures respectively.

Although constructing the interface with only a set of sensors limits the gestural vocabulary, we chose a wearable design approach over detecting in-air gestures (Godøy et al., 2005) because the wearable interface offers tangible interaction. Felt Sound’s touch and force-sensitive interaction enabled users to embody the felt experience of performance beyond localized vibrotactile feedback. In addition to amplifying the physicality of the performance (see Figure 5), it enhanced awareness and sense of the performer’s own body by drawing the performer’s attention to the interaction of fingers, the relationship between the hands, and their movement in the space. This embodiment was visually and kinesthetically communicated with the audience through the performer’s presence on stage, supporting the vibrotactile whole-body listening experience that is equally shared between the performer and the audience.

#### Results and synthesis: Shared listening experiences

The bodily listening experience significantly influences the perception of music and enjoyment of music performance. This kind of listening is provided to the audience through in-air sound vibrations that are perceived both on the surface of the body (Merchel and Altinsoy, 2014) and inside the body (Holmes, 2017). While embodied listening and felt musical experiences support hearing audiences’ music performance appreciation, they play a significant role in D/HoH individuals’ understanding of music performance.

In designing Felt Sound, we addressed diverse hearing abilities in music performance. Our motivation behind Felt Sound’s performances is to create performance contexts that not only offer shared experiences for audiences with different hearing abilities but also invite each group to experience the music from the other’s standpoint. In addition to the composition providing similar bodily listening and felt sensations being shared among all listeners, the sign-language-inspired movement-based interaction offers a musical context to the signers. In this composition, ASL signers can relate to the musical context better while non-ASL signers can understand D/HoH listeners’ experience with music. Such shared listening experience is not limited to providing another modality for sensory deprivation but extends to developing a deep listening practice for all. One objective is to include the body in listening and develop an awareness of how the body plays a role in perceiving music that is traditionally dominated by auditory stimuli. This subtle perception relies on paying attention to the felt experience of how different pitches and sounds resonate in the body—the chest, stomach, and fingertips. The felt experiences are emphasized by the visual cues that appear when the performer creates music with movement-based instruments through nonmusical gestures. Another main objective is highlighting the moments of silence in music. Because music enhances the listening experience but at the same time, it can be overwhelming for the listeners to receive constant haptic stimuli on the body, balancing the experience with silent moments and pure tactile sensations becomes an important design consideration. One of our listeners commented that this aspect influences her bodily sensations and perception of movement qualities: “The felt sound highlighted moments of silence for me more so than traditional sound. I felt light and free in those moments and active in the moments of more intense vibration.”

We also discussed the deaf participants’ musical experience and preferences in semi-structured interviews. One deaf participant with profound hearing loss defined her musical experience as isolating since she needs to obtain additional information about music through nonauditory modalities. She explained that she understands the musical content, mostly the emotional content of music, through special lighting that is mapped to musical features. She positively responded to incorporating sign language gestures into music to provide context and meaning to music that deaf listeners frequently miss. She also expressed that she “felt the sound inside” of her body in the presence of strong vibrations. Another deaf participant reported that the composition felt like “the whole room was moving.”

All listeners reported some physical sensations through high amplitude, low-frequency sounds. One participant commented on the bodily listening experience: “I felt like I was using my torso to listen to music rather than my ears. The vibration seemed to be felt in and out of the torso.” Similarly, another participant reported that they “felt like the sounds are not perceived through pin-pointed sources, but rather through the entire body.” One participant with a singing background described her listening with movement qualities. She reported that “the felt sound highlighted moments of silence for me more so than traditional sound. I felt light and free in those moments and active in the moments of more intense vibration.” Some participants shared their experience with the gestural performance as they said “The premise of the piece felt like a physical expression of music through low-frequency sounds. Combining it with gestural elements created a powerful body-to-body connection.”

The audience members commented on the relationship between kinesthetic (movement and haptic) sensation and audio-visual feedback received from Felt Sound. They further expressed how the interface and the performance affected the communication between the performer and the audience:

- “The sounds definitely embraced the bodies within the audience. … the connection was more one-to-one between the performer and myself.”
- “I felt like physical and auditory movement were definitely related and emerging from the glove as the main controller. Responsive!”

This performance-oriented user study provided promising results and future directions. Building on our research with Bodyharp and Felt Sound and their performance practices, we created collaborative, shared performance practices that actively involve the participants in the design process. Bodyharp’s exploration on discovering shared perspectives of music and movement practitioners significantly contributed to the design of Felt Sound. Similarly, creating a shared listening experience that highlights the experience of the lived body and the expressivity of body movement contributed to improved inclusion and collaboration. The next section describes the final step in creating a shared performance space for a hearing musician and a deaf dancer that was presented to a mixed audience with diverse hearing abilities.

### Touch, Listen, and (Re)Act

#### Interface design

When listening to music, the Deaf community experiences profound isolation, and its members need nonauditory modality to perceive the musical context, features, and emotions delivered in performance. Beyond listening, participating in music and dance practices contributes to their daily life by developing a better understanding of rhythm (Benari, 2014), improving communication and connection with others (Darrow, 1993, 2006), or accessing opportunities for artistic and self-expression (Benari, 2014). In this workshop series, we focused on creating a shared performance space for mixed performers and audiences that increased collaboration between hearing musicians and deaf dancers.

Felt Sound was designed as a movement-based musical instrument, primarily for D/HoH listeners. Based on our experiences from the musical performances and findings from the user studies and workshops, we developed new interfaces, including the next iteration of Felt Sound’s interface for the musician and wearable haptic modules for the dancer, and a new performance (Cavdir, 2022). We collaborated with a deaf dancer to create both the interfaces and the performance. She has experienced profound hearing loss since birth, communicates primarily with sign language, and associates equally with deaf/hard of hearing and hearing individuals. In addition to her hearing impairment, she experiences some physical limitations and uses a wheelchair both in her daily life and in dancing. As a dancer, her need for movement and bodily expression necessitated a wearable design for vibrotactile feedback.

In addition to developing wearable haptic modules, we adopted Felt Sound’s wearable interface for easier connection and mobility in the second iteration. The wearable haptic modules were prototyped for the first time in collaboration with the dancer. During this codesign process, we constantly integrated her feedback and design considerations. We developed a shared performance space with the dancer where the dancer both contributed to the artistic creation and participated throughout the design process, specifically in ideation, prototyping, and performance design stages. Her participation was crucial in the design process because her persona brought her specific requirements and needs to the forefront and because she embodies a unique set of skills and artistic practices. Although the Deaf community includes diverse hearing abilities and musical interests, through this bespoke design, we observed that she still represents many deaf artists’ musical expectations, requirements, and engagements. This collaboration enabled us to codesign a shared performance space across diverse hearing and physical abilities.

#### Design approach: Cocreating music–dance interfaces

We conducted three workshops with the dancer as we codesigned the interfaces and the performance. The first workshop introduced her to the Felt Sound research, including our inclusive design approach, gestural vocabulary, and music composition for felt experiences. Through a semi-structured interview, we discussed her experience with hearing, community associations, music, and movement. Based on our discussions, we brainstormed interface ideas in three areas: (a) dancing, (b) listening experiences, and (c) collaborative performances with musicians.

**Figure 6.**
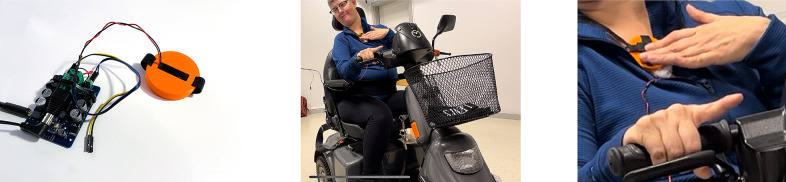
The haptic module is prototyped with a Haptuator brand actuator, bluetooth audio module, and 3D printed enclosure for the haptic module. We tested this haptic module on her chest, arms, and hands.

In the second workshop, these prototypes were tested with the dancer (see Figure 6). We also explored which types of music compositions she perceived better and enjoyed more through the haptic modules. Initially, we tested four sound files with different musical qualities, including an excerpt from Felt Sound’s previous performance, African drumming, female voice singing, and piano, using a two-subwoofer speaker system. She needed to sit close to the speakers or touch them to feel the vibrations. She reported that she felt the Felt Sound and African drumming pieces more profoundly and more nuanced than the voice and piano pieces. Although the vibrotactile listening was less pronounced in voice and piano, she was able to recognize the pitch changes and onsets in the singing and she was able to recognize the instrument in the piano piece. She was also able to feel the music through in-air vibrations. She stated that “she can feel it inside” of her body, pointing to her chest and torso. She still preferred to touch the speakers to amplify the vibrotactile feedback. Because she moves in space when she dances, she wanted wearable modules on different locations on the body: one worn on the arm and another one in the chest area. Figure 6 shows the first prototype of the haptic modules which use Haptuator brand actuator.^3^

**Figure 7.**
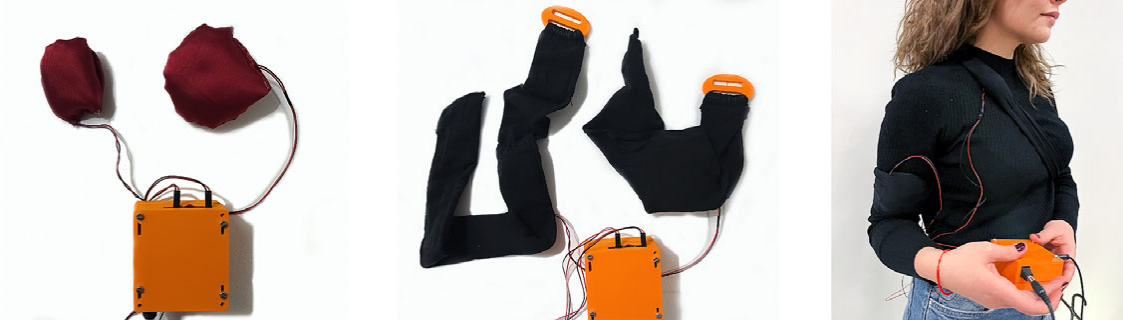
Two haptic modules, including Haptuator Mark 2 brand, type C and D actuators, are connected to the bluetooth audio amplifier. A 3D printed enclosure is designed for the amplifier and its power, audio input, and output connections. Elastic straps are designed to enclose the new haptic modules to fixture them on desired locations on the body. Fasteners are 3D printed to adjust the tightness and stability of the wearable straps.

After the second workshop, the first prototype of the haptic modules was redesigned (see Figure 7). We upgraded the haptic modules in shape and material for two reasons: (a) ergonomy and usability and (b) effectiveness of the actuation. Firstly, 3D printed modules are replaced with similarly sized fabric-foam enclosures based on the dancer’s feedback on the modules’ ergonomics. During the second workshop, the dancer commented that she needed a more flexible and softer module on her body. We also observed that she needed to hold and press the module to feel the vibrations closer to her skin. For more comfortable use, we designed both module enclosures and wearable straps that fixture the haptic modules on the dancer’s body with soft, stretchable, and stable fabric materials. Figure 7 shows the two haptic modules connected to the audio amplifier separately and embedded in the elastic straps of the wearable attachment pieces. Secondly, these interfaces were redesigned because the 3D printed enclosure failed the provide sufficiently strong vibrotactile feedback. The vibrations were more significantly perceived on the skin with a nondampening foam. The PLA material dampened some of the vibrations through its thickness and infill structure and the enclosure required a foam layer between the part and the actuator to avoid the rattling noise. Because this two-layer structure of the first prototype decreased the intensity of the vibrations and proximity to the skin, it is replaced with a fabric-foam enclosure. Additionally, because the wearable straps in the first prototype were unable to provide enough support during dancing, they are replaced by stretchable materials connected by the 3D printed fasteners.

#### Results and synthesis: Shared performance spaces

Over three workshops and a demo session, we developed a performance setting across dance and music for deaf and hearing individuals. The performance practice was shared between the dancer and the musician through (a) on-body, vibrotactile music delivered from the musician to the dancer and (b) a narrative presented with sign language gestures and choreography. This performance practice focused on the interaction of the dancer with the musician in response to the musician’s live gestural performance.

In the last workshop, the interfaces were finalized where the dancer experienced listening to the vibrotactile music both through in-air vibrations using the subwoofer array and on-body sensing using the haptic modules. The dancer and the musician also developed a movement vocabulary in response to Felt Sound’s gestures. Because the dancer preferred performing a choreographed sequence more than improvisation, the dance movements were selected from the dancer’s repertoire and mapped to the musical gestures and vibration patterns. Different gestures and vibration patterns, performed by the musician, provided movement cues for the dancer. More specifically, the dancer received the music signals through the four-subwoofer speaker setup around the performance space and with the two haptic actuators (placed on the arm and the chest). In response, she performed her dance movements as she recognized specific vibration cues on her body from haptic modules and visual cues from the musician’s gestures. She reported that in order to follow the articulations in music, she needed the vibrations to be stronger, specifically to recognize the vibration-to-movement mapping during the dance. The in-air vibrations from subwoofer speakers only provided felt sensations on the body without the nuances of the sound. Due to these significant differences between the two vibrotactile modalities, we continued our dance–music collaboration with the haptic modules, using sounds from subwoofers to support the on-body listening.

The dancer’s experience with two vibrotactile modalities showed how to better approach creating felt experiences. We observed that the subwoofer array that surrounded the room created a more embodied, whole-body listening experience that was felt in the room and inside the body. The dancer reported that the in-air vibrations were felt on her body and the surrounding space and it is a form that she was used to experience sound. The workshop setup provided more amplified sensations of sound vibrations in the room. However, in-air vibrations challenged her to detect the nuances in the music. The on-body haptic modules offered more sensitive and articulate listening experiences, especially when simultaneously worn on more than one location on the body. She explained her experiences wearing the haptic modules: “I understood what the sound was about, I could feel the difference. I could feel the diversity and the flow in music.” The results of these experiments also showed that understanding the music through different modalities of vibrations, including learning how to feel and interpret the vibrations, requires ongoing practice for Deaf users.

The same performance was tested with hearing users. One participant wore the haptic modules and preferred to close her eyes while listening. She reported that “[she] had to listen in a different way, not only aurally but also in her whole body,” describing it as an internal listening experience. The vibrations created a sensation that resembled “a dialogue between skin and heart.” Similarly, one participant with a music background said that “she did not consciously separate two different modalities” (sound vibrations from the subwoofer speakers and on-body vibrations from haptic modules), instead “[the listening] became one experience.” Another participant reported that it was effective to feel the music through vibrations first, “neutralizing the listening experience beyond localized sensations on the body.”

These reports showed that the listening experience shifted the dancers’ focus onto their bodies, “regardless of an observer” as one participant expressed her first-person experience. This approach created opportunities to share embodied listening experiences among performers, connecting the vibrations created by the music with the movements of the dancer. One hearing participant with a dance background explained her experience with vibrotactile feedback and movement as “[…] the skin felt the vibrations that went into the body and the movements grew from there.” In addition to encouraging the inner motivation of the dancers to move, one participant reported both the dance and music performance using body movements and the sign language gestures expressed a connection, exuding dance-like qualities.

**Figure 8.**
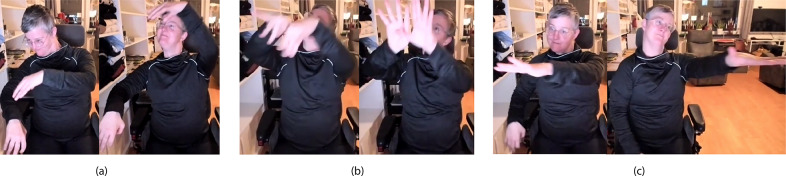
The deaf dancer composed four dance movements in response to the different vibration patterns. Three movements are connected with arms waving gestures in the air to represent the intensity of the vibrations and inspired by dance metaphors representing (a) raindrops with the dabbing gestures, (b) flying bird by flexing the fingers’, and (c) horizon by scanning space with the hand and the gaze.

**Figure 9.**
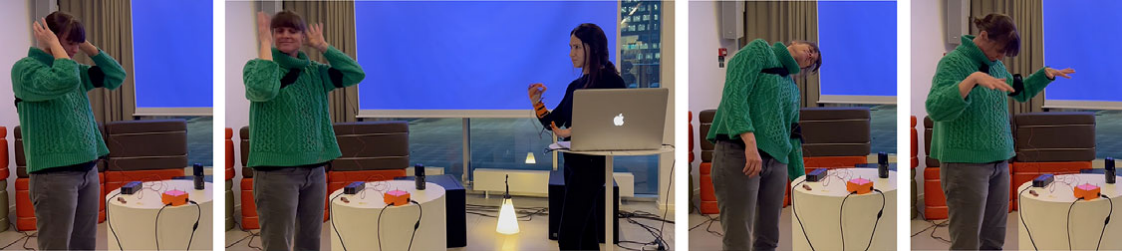
The embodied listening experience where the sound is delivered by the haptic modules combined with the four subwoofer speaker arrays was also tested with a hearing dancer who improvised dancing to the music performed by the ASL gestures.

In addition to sharing the performance experience through felt sensations on the body, we cocreated a shared gestural vocabulary. A dance movement vocabulary was developed based on the connection between the musical gestures, their sonic response, and vibrations. Due to COVID-19 restrictions, the documentation of the collaborative performance was recorded virtually. Figure 8 shows some of the gestures composed by the Deaf dancer in response to Felt Sound’s gestures, sounds, and vibrations. She created a narrative choreography following a storyline of a storm where different parts of the story were associated with the vibration patterns and intensities. In Figure 8a, the dabbing gesture over the hand corresponded to the slowly increasing intensity of the vibrotactile sound waves, matching with Felt Sound’s *discover* gesture; Figure 8b shows the joint hands with fingers imitating a flying bird, representing the pulsating effect on the sound and vibrations, and finally the scanning gestures with one hand and the gaze in Figure 8c completes the performance as the sound and the vibrations fade out. These three dance movements are connected with the movement of arms waving in space, representing the high volume and frequency of the vibrations. Additionally, the *poetry* gesture in Felt Sound provided a movement cue to the dancer to change her location on the stage. Figure 9 shows the improvised movements of the hearing dancer while she listened to the music both with the subwoofers and the haptic modules on her body.

## Emerging Themes and Practices

Throughout our design and performance process, we collected participants’ experiences with the movement-based interaction through interlaced body- and music-based exercises (such as the learning process with the Bodyharp or developing music-haptics-movement mappings in Touch, Listen, and (Re)Act), embodied listening spaces and practices (such as the immersive and haptic performance space in Felt Sound and dual-modality haptic listening in Touch, Listen, and (Re)Act), and participation in cocreating music-movement mapping (such as collaborative performance design with music and dance gestures in Touch, Listen, and (Re)Act)) (Cavdir, 2022). Each design approach specific to individual case studies informed the design, performance, and participation practices of the next study. These practices provided tools to aesthetically articulate musical and gestural expressions and to reflect on the relationship between the two. This evaluation process based on creative artifacts would be impossible without integrating movement-based music performance into the design cycle.

Both by conducting thematic analyses and aesthetically evaluating the process of creative practice, we derived two overarching themes and one inclusionary performance practice. The following subsections present the results of these thematic analyses that were accessed through the aforementioned tactics: defamiliarization, extending musical movement vocabulary, and creative artifacts (section “Design Approach and Methodology”). These themes and practice commonly emerged across three case studies toward integrating movement-based musical interaction into inclusive design practice for increased collaboration across diverse hearing abilities.

The defamiliarization of both music-making with wearable interfaces and nonaural, bodily listening revealed shifting awareness of participants to the moving body and the listening practice. Extending the musician’s gestural and movement vocabulary emphasized the bodies’ active participation in the musical expression and in listening not only through aural channels but through kinesthetic interaction. Creating design and musical artifacts as a research outcome highlighted not only learning the musical interfaces and their sound-to-movement mapping but also articulating such embodied, first-person experiences through a creative practice by composing musical and movement statements.

### Thematic Analysis

The interview and questionnaire data collected in the three case studies were independently analyzed. These experiments collected self-reported experiences and reflections of the participants/performers (in the first study), the audience members and the performer (in the second study), and the codesigners (in the third study).

After within-case analysis for each study, a thematic analysis followed across the three case studies. Two common themes emerged through cross-case analysis:

- Awareness on the moving body that emerged through the connections between (a) body-to-instrument, (b) body-to-body of the performer and the audience, and (c) body-to-body of the coperformers.
- Awareness on the listening practices that utilize some of the soma tactics and body movement throughout the experience.

Table 6 presents some of the related themes and associated keywords and codes from the within-case thematic analyses. Some of the individual case study themes are common to all studies and some of them are combined to overall emergent themes (see themes highlighted in bold in Table 6). For example, themes such as *sound awareness*, *bodily listening*, and *listening awareness* are combined under *awareness on listening.* The comparison and contrast on these same or similar themes across case studies can be observed in Table 6. For example, similar experiences such as *listening with the torso* or *in whole body* are reported in different case studies.

**Table 6.** Some of the common or related themes from within-case thematic analysis

Case study	Keywords/Codes	Themes
Case 1	Learning how to compose and perform with, experience of discovering, intuitive	Learning process
	Becoming more aware of sounds, attentive listening	**Sound awareness**
	Embodiment, sounds come from movements, feel more in my body	**Movement awareness**
	Explore sonic space while moving body in physical space, freedom in space by movements and by the music	**Music and movement connection**
	More about the relationship with the instrument, heavy-light qualities in the arms, attached to arm	**Body-to-instrument connection**
Case 2	Functional meaning, meanings of gesture, knowledge of ASL	Meanings and metaphors
	Touch, tactile/auditory change	Tactility of interaction
	Listening with the torso, feeling through the entire body, in and out of torso	**Bodily listening**
	Relation between physical and auditory movement, mapping, gestures’ correspondences with sounds	**Sound and movement connection**
	One-to-one connection with the performer,	Shared listening
	Highlighting silences, movement and sound qualities, vibrations, immersive sound sources, listening through body	**Listening awareness**
Case 3	Not only aurally, in whole body, feeling on skin, beyond localized sensations on body, feeling through vibrations, inside the torso	**Bodily listening**
	Understanding sound, diversity and flow in music, listening as one experience, listening in a different way	**Listening awareness**
	Movements emerge from vibrations felt on the skin, exuding dance-like qualities, cocreating the choreography	**Movement awareness**
	Communication between the musician and dancer, cocreating the choreography, learning of the mapping	Codesign and shared practice

*Note.* The themes highlighted in bold shows the contributing themes in the cross-case analysis. The content in the table shows only the most relevant themes and codes to all three studies.

#### Awareness on the body

As previously described, the moving body is integral to the design of movement-based musical interfaces that positions one’s own bodily and felt experience at the center of musical expression. Our case studies demonstrate how wearable interaction supports first-person perspective through felt experiences, from building body-to-instrument connection and attachment to sensing music directly on the body.

##### Body–instrument connection

The studies with the Bodyharp showed that the participants developed an increased awareness or attentiveness to their moving body, specifically through the wearable connection. One participant expressed that “the Bodyharp made me feel more in my body” and explored “How do I move that makes a sound?” Another participant approached this relationship with a different perspective by comparing her experience dancing with musicians and emphasized that “Bodyharp was following my body” more than her following an external sound source.

Participants emphasized how the connectivity between the body and the instrument challenged the dualistic view of the mind and body during the interaction. One participant stated that “I never thought of myself in the process. It wasn’t about me, it was about the relationship with the instrument. I was a different person with this instrument.” Similarly, most participants related this extension of the self in relation to their kinesphere. One participant reported that since movement is an integral part of the interaction, “the instrument made me think more about, […], [the instrument] as an interface to the space or the potential for movement. The instrument is the entire called space around that object!” Another participant highlighted how the interaction affected the body’s spatiality: “My sense of self was expanding, so it was easier to use the space.” Similar experiences of expanded space by both movement and sound were expressed: “The use of space felt very expansive, and the sound carried through my body.”

##### Body–body connection between performer and audience

In Felt Sound, the audience experienced a bodily connection to the sonic interaction through sound in space and the performance’s visual and gestural feedback. One participant shared their experience with this embodied composition: “The felt sound highlighted moments of silence for me more so than traditional sound. I felt light and free in those moments and active in the moments of more intense vibration.” In addition to the sound composition, one of the strongest contributors to this bodily connection was the gestural performance. One participant commented on this physicality: “The premise of the piece felt like a physical expression of music through low-frequency sounds. Combining it with gestural elements created a powerful body-to-body connection.”

This body-to-interface relationship was also carried to a bodily connection between the performer and the audience. One participant reported their experience: “I felt like the sounds are not perceived through pinpointed sources, but rather through the entire body. The sounds definitely embraced the bodies within the audience. However, rather than feeling connected with other members of the audience, the connection was more one-to-one between the performer and myself.”

##### Body–body connection between performers

In Touch, Listen, and (Re)Act, both the haptic feedback and the closely linked mapping between musical gestures and dance gestures supported a body-to-body connection between the two performers. The audience from the public performance reported that one of the most effective parts in the performance, based on the excerpts of the music-movement composition and the live performance, was “the dialogue between the musician and the dancer.” For one participant, both body movements and sign-language-inspired gestural performance exuded a dance quality. Between the dancer and the musician, wearable haptic modules provided the musician’s sonic expressions directly to the dancer. The process of cocreating a movement composition also supported them to explore how one gestural expression related to the other in a way that requires constant listening to each other’s body movements.

#### Awareness on the listening

With Bodyharp, the participants’ focus shifted to the sonic outcome of their movement, making them feel less self-conscious. One participant with reported that “most of my attention is when I do something [a movement] on how the sound comes out.” Another participant shared similar experiences with focusing on the sound-creation process by saying, “The sound carried through my body […], I also felt very focused […]. There was an interesting process of attentive listening.” One participant commented on the composition practice with Bodyharp. “being asked to create [a musical statement] makes me much more aware of the sounds that my movements are creating.”

According to all participants in the Bodyharp study, the instrument encouraged them to move more while creating music and be more attentive to their body and body movements. For example, one participant highlighted that “the interaction between what you do and what you listen [to] really forces you to be really engaged and in tune in your movement as a source of inspiration.” Similarly, another participant reported that when she “felt out of control of the instrument,” she moved back to “listening to, thinking in terms of gesture, and feeling how to play, and letting go of the idea of always knowing how the instrument will sound.”

The participants’ responses to their listening experience highlighted how interconnected the awareness was built on the moving body and the listening. The Felt Sound study more specifically focused on shifting the listening experience to a more bodily experience using kinesthetic music composition. Participants used their bodies more to listen; in other words, they increased their awareness of how much the body was involved in the listening. One participant stated that “I felt like I was using my torso to listen to music rather than my ears. The vibration seemed to be felt in and out of the torso.” A similar experience was accessed in the Touch, Listen, and (Re)Act study. Hearing participants reported that they “felt it more than hearing [the music].” Similarly, one participant shared her experience of feeling the music “inside of [her] body […] in the torso and entire body.”

In the second case study, the biggest limitation was the lack of deaf and hard of hearing participants. Although, the study participants had experience with ASL performances (such as performing arts and new music, song interpretations, or theater), the study fell short in collecting the listening experiences of D/HoH members due to resource demanding process of reaching out to the D/HoH community. However, we were able to collect feedback on the listening experience with Felt Sound with one Deaf participant. Comparing the responses from both the second and the third case studies and from both hearing and deaf participants revealed how assistive wearable instruments and music performing and listening systems can support the experiences of hearing listeners. In the public performing session, we collected hearing participants’ experiences with the two haptic modalities.

For many, haptic modalities provided a listening experience outside the familiar. Such defamiliarization supported their increased attention on the bodily listening. They reported that they had to “listen in a different way, not only aurally but also in [their] whole body,” and they did not “consciously separate two different modalities” (sound vibrations from the subwoofer speakers and on-body vibrations from haptic modules), instead “[the listening] became one experience.” Exploring such an embodied form of listening expanded participants’ movement vocabulary in musical interaction. One participant highlighted how she freely moved while listening through both in-air and on-skin haptics as “[…] the skin felt the vibrations that went into the body and the movements grew from there.” The vibrations created a sensation that reminded her of “a dialogue between skin and heart.”

Additionally, bodily listening increased participants’ attention to musical qualities such as silences or intensities of sound effects. According to P5 in the Felt Sound study, the silences in sound and movement amplified the correspondences between music and gestural performance. These changes in silences and intensity of the vibrations were felt on the body. This feedback was reported in the third case study by the Deaf participant. She repeatedly reported the silences in vibrations and interpreted some of the low vibrations as silences. The level changes and varying amplitude envelopes were These similar responses from both participants with and without hearing impairments can inform the sound composition when designing bodily listening experiences with haptics. One of the limitations we experienced in the third case study was the time constraints for the participant to learn how to listen through haptics, considering that she had little experience listening to music. It highlights the need to develop a practicing framework to learn listening through haptics and how the vibrations are correlated to musical features. The same approach can apply to composition practices. For composers and designers, a framework to design music-vibrotactile mapping for increased awareness on the listening can further be studied.

### Performance Practice and Aesthetics of Interaction

As a creative practice, embodied musical interaction offers opportunities to integrate creating music and movement artifacts into learning the interaction and exploring affordances within the limitations of wearable interfaces. When the first case study asked participants to create both musical and movement compositions, the participants were able to approach music-making from a perspective of designing through kinesthetic experience. Introducing developing creative artifacts as research outcomes supported participants’ intentionality. For example, one participant reported that “I become much more aware of the sounds that I am creating, *not so much of exploring but composition*, having repetitions, going back to the themes I had, and repeating the same movements with my body.” Another participant explained that “[composition and movement improvisation] captured this intermediate explicit type of my movement and the sound patterns that I create.”

The second case study was based on creating a performance space, including an inclusive composition, a gestural performance, and a mixed audience. For the performer, creating musical statements that were also received through the felt experiences was both a result of the interaction and an inspiration for the experience. It revealed unexplored felt experiences of listening and future directions for inclusive composition. Some participants reported that the context of using another language (ASL) enriched their experience: “[…] knowing that it is based on ASL gave it a special meaning, without realizing what it meant,” “The gestures seem meaningful even without ASL knowledge,” and “ASL brings more context to the gestures and I interpreted them as metaphors rather than indexical references to their functional meanings.”

Similarly, the choreographic narrative in the music and dance gesture mapping offered a shared context for coperformers in the third study. This study’s collaborative performance design across performers with different hearing abilities made understanding of music and movement relationship a process-oriented practice. This practice led to a transformation of new listening ideas and gestural connections between two artistic disciplines. For example, the Deaf codesigner’s listening experience in the third case study showed new directions of designing for experiencing, highlighting her unexplored needs in music collaboration. As she stated, “This is the first time I can listen to music and I need to learn and practice what causes the changes in the music.” At the same time, the collaborative performance enhanced her practice in understanding “the diversity and the flow in music” through codesigning a dance narrative based on vibrotactile stimuli of music.

## Discussion

We investigated how wearable instrument design influences our embodied and inclusive design approach: cocreating felt experiences, sharing embodied listening practices, and performing to evaluate. First, our studies showed that the kinesthetic sense and expression significantly contribute to the felt experiences of music performance. Movement-based musical instruments both encourage performers’ participation in creating music and movement compositions and support performers to express musical ideas with their body movement by focusing on the music-movement relationship. Second, WT supports developing more inclusive musical instruments that provide opportunities for collaboration across diverse abilities. Third, performance as a creative artifact is an integral part of the evaluation of the instrument design process.

### Felt Experiences

Sheets-Johnstone emphasizes the felt qualities of the moving body perceived through kinesthetic sense (Sheets-Johnstone, 2011). As Loke (2009) states, the moving body is essential to our experiences, along with our primary senses, emphasizing “the instrumentality of the body” (Merleau-Ponty, 1962). In our first case study of movement-based musical instrument design and performance, we discovered that performers were able to integrate their bodies into the musical instrument with Bodyharp, using the body as the instrument. Their body movements were no longer an external expression but a tool to create music while simultaneously responding to the instrument. We discovered that the performers approached the instrument with its own agency until they were familiar with the bilateral reaction between the instrument and themselves. The primary reason behind their approach was that the wearable interface constantly detects the motions of the performer and provides unexpected musical events until the performer becomes aware of the living interaction between their body and the instrument.

Both Bodyharp’s wearable interface and music-movement affordances highlighted the performer’s kinesthetic expression. We observed such expressions in performers’ musical and nonmusical gestures during the user studies. In addition to performing with body movements to *create* music and *control* the sound parameters, the performers interacted with the instrument using *ancillary* gestures that had no contribution to the sound (Godøy and Leman, 2010). These nonmusical gestures revealed performers’ inner intentions of bodily and movement expressions. We found that the movement-based musical interaction allowed performers to explore the nuanced balance between controlling sound and expressing musical ideas with ancillary movements, reflecting their inner response. As many participants reported, creating music through bodily felt interactions encouraged body movement without worrying about the third-person perspective, increasing their focus solely on sound and movement. Sheets-Johnstone (2020) calls this “hereness” of the lived body, stating that “the experience of hereness is a felt bodily presence.”

Such kinesthetic sensations were not limited to body movement but are supported by tactile interaction and active and passive haptic feedback. In Bodyharp, these interactions were provided through the instrument’s affordance to draw connections between the performer’s body and kinesphere using the sting attachment (Laban and Ullmann, 1971). This on-body interaction with the strings provided passive haptic feedback that supported the body-half connectivity (Bartenieff and Lewis, 1980; Bartenieff et al., 1984), which increased body awareness and encouraged new interaction. In addition to the strings, the force-sensitive interaction provided opportunities to sonify different aspects of movement qualities and the inner attitude of the mover (Laban and Lawrence, 1974). We discovered that mapping between tactile sensors and the sound effects supported music-making more intuitively–“tuning into the instrument.” Incorporating the movement qualities into music-making encouraged performers to follow their inner attitude, helping them simultaneously stay present and respond to their movement. Similarly in Felt Sound, the tactile interaction indicated the source of the sound creation (wearable sensors capturing the hand and finger movements) and amplified the sound to movement mapping while gestural elements (sign-language inspired movements) created a body-to-body connection between the audience members and the performer. While the movement-based performance enhanced the connection between the audience and the performer, addressing diverse hearing abilities supported the shared felt experience among the audience members.

When movement interaction is involved, the nature of the design outcome requires an experience-based approach. Throughout three case studies, we studied different aspects of felt experiences such as movement expression for the performer, embodied listening experience for the audience members, and sensory replacement with the support of on-body, felt interactions. Among three instruments, Bodyharp highlighted how to approach the body as the instrument and the design practices to incorporate performer’s bodies. Felt Sound created shared spaces for more embodied listening experiences. The felt experience from in-air haptic sensations contributed to the lived bodily experience in music but also it offered embodied listening practices beyond sensory ideals. Touch, Listen, and (Re)Act actively combined on-body felt experiences of music-making and listening as an embodied practice and involved participants as designers in the process.

### Embodied Listening

Embodied listening offers an experience beyond nonaural means of musical expression. However, decoding which musical features are perceived through hearing or through the body remains unclear. Felt Sound’s performance with low frequency, high amplitude music composition redirects the audience’s attention from solely auditory listening to more tactile, visual, and kinesthetic on-body listening. Drawing from Deaf people’s lifelong engagement with bodily listening, we observed that hearing people benefit from more embodied listening experiences just as much as D/HoH individuals.

First, most participants realized the felt sensations of the sound on their bodies. Although the Deaf listeners previously experience music’s vibrotactile feedback through the loudness of music, specifically of the beats and through listeners’ proximity to the sound systems, they described listening to the Felt Sound’s performance as feeling the sound “inside their body.” Without touching the speakers, two on-body sensations were reported: in the torso and in the entire body. When the listeners touched the speakers as they listened, the vibrations were perceived more dominantly on the fingertips. We found that the composition significantly influenced where the sound is felt on the body. Although the exact localization of vibrotactile sensing falls outside the scope of this research, among both normal hearing and D/HoH listeners, we observed that “the sound vibrations [were] felt in and out of the torso” (Cavdir and Wang, 2020). For the sound to be felt in the body, the intensity of modulation needs to be higher and changes in musical events need to be more pronounced. Where the composition falls short in delivering on body sensations, the hands or fingertips perceive more nuanced changes. These sensations can be amplified with wearable haptic actuators, specifically when multiple devices are coupled at different locations on the body.

Second, the perception of movement qualities of the performer’s gestures significantly contributed to the listening experience. Unlike in the nonvibrotactile compositions, the audience perceived the movement qualities of the performer’s gestures in Felt Sound based on the intensity and rhythm of the vibrations. The moments of vibrations and silences were associated with heavy or active and light or free movement qualities, respectively. For D/HoH listeners, listening with visual cues becomes an important aspect of embodied listening and extracting the emotional information of music. In addition to the haptic sensations of the vibrotactile movement-based music, the visual and kinesthetic components contribute to developing an embodied listening experience. Because embodied listening “involves an implicit learning” (Leman et al., 2009), the associations and correspondence between music and both movement and vibration patterns require longer practices. Music performances benefit from incorporating embodied listening practices that support felt experiences.

Leman et al. (2009) discuss how embodied listening relates to listeners’ subjective understanding of perception and action and how its perceived expression can be shared by a community. Another finding from the listening experiences among mixed audiences is rooted in the shared aspect of listening that offers both equal entry points to music performance and opportunities for collaboration. In Felt Sound, we discovered the physicality of performance and felt sensations of music established a body-to-body connection between the performer and the audience members. Similarly, in the Touch, Listen, and (Re)Act, because the musical information and agency are shared between two performers, this connectivity necessitates constant listening using visual and tactile feedback. Additionally, the movement-based performance delivered the kinesthetic aspects of such embodied listening experience both to the musician and dancer. Their bodily movement expressions both responded to each other’s movement performance and contributions to the sound. We found that embodied practices highlighted the first-person experience of both the performer and the listener while sharing these experiences provides a social connectedness that is both integral to music expression and embodied listening.

### Future Directions in Inclusive Design

#### Qualitative analysis of wearable instruments

A qualitative evaluation of our movement-based wearable designs provides the strongest fit for our design considerations and for collecting users’ insights and lived experiences. To study specific performances and use cases of wearable instruments, our motivation critically incorporates theoretical grounding from embodied musical interaction, somaesthetics and movement-based interaction, and inclusive design research. The qualitative analysis supports three core aspects of our research: (a) designing creative artifacts as research outcomes, (b) verbalizing tacit experiences, and (c) incorporating artistic context and behavior into the design process. These qualitative methods provide a strong fit not only for practice-based, artistic research but also for engineering and design studies (Szajnfarber and Gralla, 2017).

- *Creative artifacts as research outcome:* Candy and Edmonds (2018) characterize the creative practice “not only by a focus on creating something new but also by the way that the making process itself leads to a transformation in the ideas” that contributes back to the creative artifacts. Similarly, our primary research focus includes both the movement-based musical interfaces and their design and performance practices. As creative artifacts, new musical instruments, lead to new artifacts, music composition, and performances. Because of this close link between the instrument design, performance practice, and research assessment, a qualitative and exploratory evaluation is needed. Such evaluation offers distinct advantages to study creative artifacts and the insights and reflections from their creation process: “the possibility of taking account of context” such as the inclusion of hearing impairments, the ability to describe the study as it is perceived from different observer perspectives such as combining first and third-person perspectives, and the “strong process orientation” such as learning and creation processes (Glaveanu, 2010). Additionally, we observed that the movement-based musical instruments provided musicians and movers with bodily interactions that uncover some subjective but shared correspondences between music and movement. Ramsay and Rockwell (2012) state that creative artifacts are “tools that show us something in a new light.”
- *Tacit experiences that are difficult to verbalize:* Because the lived and movement-based experiences are often challenging to verbalize (Moen, 2006; Loke, 2009), such qualitative and explorative evaluation reveals unexplored intentions and future directions. Articulating felt experiences requires “a shift in our perception of design […] toward framing design as a holistic body–mind practice […]” (Sas, 2019). A practice centered around creating and performing while experiencing felt dimensions of music-making offers verbal and nonverbal ways to capture and articulate such tacit experiences. For example, we observed that asking users to compose with body movements and musical interfaces and later choreograph with free movement provided an effective qualitative assessment that led users to develop a movement vocabulary for articulating their felt experiences.
- *How artistic context and behavior influence the design process:* We realized that users’ insights and reflections are more significantly revealed during collaborations and performances. For example, Bodyharp’s performance in the same performance space with dancers showed how performers can collaboratively interact with the instrument. It offered choreographic cues for dancers. These experiences were integrated into the design of Touch, Listen, and (Re)Act, for a collaborative performance between a musician and a Deaf dancer, contributing back to the design ideation and decision-making steps. Such collaboration and performances offered important research outcomes that reshaped how we constructed the wearable interfaces and our approach to DMI design. We realized that our design processes significantly benefited from (a) defining the artistic context in which the artifacts will be used, (b) integrating performance into the design loop, and (c) observing collaborator’s interaction and artistic behaviors with the artifacts.

#### From user-centered design to participatory design

In our earlier studies of movement-based musical instruments and interactions, specifically with Bodyharp, we focused on incorporating the user feedback iteratively into the design and composition of the instrument. In Felt Sound, we asked the audience to report their listening experiences and embodied explorations of their connection to the performer and other audience members. Both these studies encouraged lived experiences of the users; however, the designer/researcher and user roles remain divided. Such roles merged when the designer performed with the instruments by herself, utilizing performance as an evaluation method and emphasizing the first-person experience (Varela and Shear, 1999; Engelsrud, 2005). Although we incorporated users’ feedback into the next iterations of musical interfaces, we critically analyzed users’ reflections on the whole performance/listening experience beyond product development.

In addition to collecting user feedback, we structured the user studies so that the research outcome and the analysis lead participants to construct creative artifacts (compositions, choreographs, and self-reported experiences; Bossen et al., 2016). This method enables us to shift the evaluation of instruments and the design processes from the third-person to the first-person analysis. In exploratory and semi-structured qualitative evaluations, we found that the participants’ creations—in music and movement forms—reveal inner attitudes and lived experiences beyond observing and discussing. While performance created an evaluation opportunity for the instruments in action, performers’ insights from making, reflecting, and evaluating fed back to the artifacts (movement-based musical instruments) themselves. By incorporating music and movement composition into more traditionally designed user studies, we were able to encourage user participation and collect their first-person experiences.

Compared to the user-centered practice with Bodyharp and Felt Sound, the participatory design process in Touch, Listen, and (Re)Act significantly highlighted the first-person perspective of the performers in two ways: cocreating (a) music-dance instruments and (b) performance spaces both for collaborative dance and music practice and for different hearing abilities. This study combined both user-centered methods such as generative ones (e.g., brainstorming) and participatory design methods such as cocreation and collaborative performance. We found that an inclusive design approach invites participatory practices. At the same time, the participation of users with hearing impairments in the design process contributes to more inclusive research outcomes. Performance as both a design step and an evaluation method opens up the possibility to address the challenge of validity in first-person research (Sas, 2019). Moving forward, we will leverage performance and collaboration opportunities in this shared space among people with diverse abilities.

## Conclusion

Through the lens of movement-based musical interaction design, we uncovered the implications of embodied practices for more inclusive collaboration with artists from different disciplines and with diverse hearing abilities. Movement-based wearable musical instruments and their design practices combine somaesthetics, embodied musical interaction, and felt experiences. The emphasis on the lived experiences highlights the performers’ first-person perspective in the design and evaluation processes. At the same time, this consideration provides us with the opportunity to involve performers in several design steps. Especially when working with hearing-impaired performers, their participation is crucial to not only address their specific skills and needs but also create a shared performance space. These bespoke designs impact the Deaf community beyond individuals, removing some of the barriers to actively participating in creative practices such as music and dance performances.

Results, insights, and reflections from each project contributed to the next one’s design. From our studies on wearable instruments that capture nuanced musical gestures and expressive body movements, we found that this dual interaction, combining body movements from dance and music, provides more equal entry points to music-making. We incorporated our results and observations on designing bodily expressions and felt experiences with Bodyharp into Felt Sound’s music performance for a shared audience of D/HoH and hearing individuals. This project revealed the need to actively collaborate with hearing-impaired individuals and more closely engage with Deaf communities. When we designed instruments specifically tailored for a Deaf dancer in Touch, Listen, and (Re)Act, we observed how participatory design can highlight incorporating the performer’s first-person experience into designing DMIs. We cocreated a performance context where both the felt experience of creating and listening to music and the performance with body movements were shared.

Moving forward, we plan to explore new participatory design practices to involve Deaf artists in music performance. The inclusive design practices and participatory approaches for sound interaction and instrument design still lack a framework that provides guidance to music designers, performers, and researchers. One of the challenges in these practices is the difficulty of accessing the Deaf community members who might benefit from embodied listening and music-making tools. By offering a more shared performance space for D/HoH and hearing individuals, we hope to engage with more Deaf artists. We believe that in addition to providing tools for embodied listening and performing opportunities, creating shared performance spaces increase the participation of a more diverse user group of performers, designers, and musicians.

## Data Availability

The data that support the findings of this study are available on request from the corresponding author, D.C., on limited basis. The data are not publicly available due to restrictions defined by Stanford IRB approvals for nonmedical human research and for protection of the privacy of research participants. All code created for this study is available upon reasonable request under Creative Commons Attribution 4.0 International License.

## Footnotes

1
https://chuck.cs.princeton.edu/.

2
https://www.sharemusic.se/.

3
http://tactilelabs.com/products/haptics/haptuator/.
